# Potential of Artesunate in the treatment of visceral leishmaniasis in dogs naturally infected by *Leishmania infantum*: Efficacy evidence from a randomized field trial

**DOI:** 10.1371/journal.pntd.0008947

**Published:** 2020-12-18

**Authors:** Hacène Medkour, Idir Bitam, Younes Laidoudi, Ismail Lafri, Abdelaziz Lounas, Hamza Karim Hamidat, Abdeslam Mekroud, Marie Varloud, Bernard Davoust, Oleg Mediannikov

**Affiliations:** 1 IHU-Méditerranée Infection, Marseille, France; 2 Aix Marseille Univ., IRD, AP-HM, MEPHI, Marseille, France; 3 PADESCA Laboratory, Veterinary Science Institute, University Constantine 1, El Khroub, Algeria; 4 Aix-Marseille Univ, IRD, AP-HM, SSA, VITROME, Marseille, France; 5 Superior School of Food Sciences and Food Industries of Algiers, Algeria; 6 Institute of Veterinary Sciences, University of Blida 1, Algeria; 7 Laboratory of Biotechnology related to Animal Reproduction (LBRA), University of Blida, Blida, Algeria; 8 Department of Biology, Faculty of Sciences, University of Boumerdes, Algeria; 9 Ceva Santé Animale, Libourne, France; KU Leuven, BELGIUM

## Abstract

Leishmaniasis is among the world’s most neglected diseases. Dogs are the main reservoirs/hosts of *Leishmania infantum*, causative agent of both canine and human visceral leishmaniosis. Canine leishmaniasis (CanL) represents a public health problem as one of the most prevalent zoonotic diseases worldwide. Current therapeutics present drawbacks; thus, there is a need for more effective, safer, and cheaper drugs. The aim of this study was to evaluate and to compare the efficacy of oral administration of artesunate or meglumine antimoniate/allopurinol in dogs with clinical leishmaniasis. Forty-two dogs with naturally occurring clinical leishmaniasis were included in this open-label, simple randomized positive-control clinical field trial with 6 months of follow-up. Dogs received meglumine antimoniate 100 mg/kg/day and allopurinol 30 mg/kg/day for 28 days (control group, n = 26) or artesunate 25 mg/kg/day for 6 days (test group, n = 16). The animals were evaluated for their clinical evolution, parasite load (by qPCR) and humoral response at different time points: 0, 30, 90, and 180 days after treatment. Data analyses showed a significant improvement in both groups in clinical scores, parasitemia and antibody titers after treatment. Compared to the control group, the artesunate group showed significantly lower clinical score (P = 0.0001), lower parasitemia (P = 0.0001) and antibody titers after 6 months of follow-up. Compared to baseline values, a rapid, significant reduction (P < 0.012) in antibody levels, 2.28- *versus* 3.04-fold for the control *versus* artesunate groups, respectively, was observed 30 days after treatment. Antibody levels continued to decrease further in the artesunate group, where 58% of cases became seronegative at the 6-month follow-up. All qPCR-positive dogs were negative after treatment with artesunate, while 14.3% remained positive with the appearance of two new cases in the control group. Artesunate was well tolerated, and no side effects were recorded. Treatment failures were similar in both groups with 27.27% (6/22), including 18.18% (4/22) mortality in the control group, *versus* 26.66% (4/15), including 13.33% (2/15) mortality in the artesunate group. This is the first report showing the potential of artesunate in the treatment of dogs with clinical leishmaniasis. Artesunate showed higher efficacy than the current first-line treatment for CanL without any adverse effects. It could be a good alternative chemotherapy for CanL, and may be considered for further studies in human leishmaniases. Further clinical trials are needed to confirm these findings, to determine if there are relapses after treatment and if dogs remain infective to sandflies, to define the ideal therapeutic dosage and duration of treatment with artesunate.

## Author summary

Canine leishmaniasis (CanL) is a fatal, zoonotic vector-borne disease caused by *Leishmania infantum*, a common pathogen for both humans and dogs. Most CanL therapeutics are toxic, expensive, or ineffective. Artemisinin and derivatives have recently demonstrated potent antileishmanial activity in vitro and in experimental models. In this study, dogs with clinical leishmaniasis were randomly included in one of the treatment groups: meglumine antimoniate/allopurinol (control) or artesunate (alternative). Dogs were followed up for 6 months for their clinical score, parasitemia and *Leishmania* antibody levels. Both groups showed improved clinical scores, parasitemia and antibody titers after treatment. After six months of follow-up, treatment success was very similar in both groups, and 72.73% (16/22) of the controls *versus* 73.34% (11/15) in the artesunate group had clinical improvement. All dogs initially seropositive by PCR became negative after artesunate treatment, while 14.3% remained positive with the appearance of new cases in the control group. Antibody titers decreased rapidly (from day 30) from baseline especially in the artesunate group, where 58% of the dogs converted to seronegative after 6 months. Artesunate could be a good alternative for treatment of leishmaniasis. Additional clinical trials are needed to obtain more data on this drug.

## Introduction

Visceral leishmaniasis (VL) is an infectious, vector-borne, chronic, systemic disease. It is a major zoonosis with significant clinical and epidemiological control priority in the world. The domestic dog is the main animal reservoir, while other wild animals, such as foxes, play a role in sylvatic transmission. *Leishmania infantum* (syn. *L*. *chagasi*) is the causative agent of both human VL and canine leishmaniasis (CanL) [1]. In the last few decades, epidemiological changes in VL, including increases in incidence and mortality rate and its spread to new and even urban areas, have been observed [2–7]. As the dog is the main reservoir for human infection, there is a significant overlap between areas of high prevalence of CanL and human mortality attributed to this parasitosis [8,9]. Among the reasons led to its spread: the lack of vaccines, failures in controlling vectors and the increasing selection of drug-resistant parasites [10].

CanL is a severe disease that affects several million domestic dogs in countries on both sides of the Atlantic Ocean (mainly Europe and South America, but spreading in Africa and Asia as well) and may kill infected dogs when left untreated [11,12]. In Mediterranean countries, where infection rates are up to 63% including at least 2.5 million seropositive dogs [13–15], CanL represents one of the leading causes of death in dogs [16,17]. It is a growing disease that has become one of the most important canine diseases imported to Central Europe [18]. North Africa is highly endemic and important prevalence of CanL were reported in Algeria [19,20], Tunisia [21,22] and Morocco [23]. In addition, *L*. *infantum* has been well described in wild animals in Algeria, such as the jackal [24].

Clinical manifestations in *L*. *infantum*-infected dogs are very wide and variable. Consequently, several CanL forms can be typified by their numerous clinical signs, including cutaneous, mucocutaneous and visceral forms [25]. In addition, *L*. *infantum* infection in dogs can manifest as subclinical infection, a self-limiting illness or a serious life-threatening disease [26].

Treatment of CanL is a challenge because of the intracellular localization of the parasite [27]. Moreover, the World Health Organization (WHO) has suggested reserving antileishmanial drugs used for human VL for exclusive use in human leishmaniasis and not for veterinary medicine because of suspected drug-resistance development from use in animals [20]. Despite this, the first-line treatment for CanL is currently the combined use of leishmanicidal agents used as second-line drugs for humans (e.g., pentavalent antimonials, miltefosine) and allopurinol [25,28–31]. Some of the chemotherapeutic compounds used for CanL are included within the 19th edition of WHO Model List of Essential Medicines (April 2015) against leishmaniasis: pentavalent antimonials, miltefosine, amphotericin B deoxycolate or liposomal formulations, and paromomycin. However, some other drugs are also effective, such as allopurinol, pentamidine, enrofloxacin, marbofloxacine, metronidazole, spiramycin, and ketoconazole [32]. Determination of the best drug is based on clinical examination and staging of CanL, according to immunodiagnostic test results, clinical signs, clinicopathological abnormalities and parasite load [29,25]. Most of these treatments are expensive and do not achieve complete cure of the disease, and some of them can cause important side effects [31,33]. Vomiting, lethargy, diarrhea and nephrotoxicity are common during the current treatments with pentavalent antimonials and miltefosine [27,31,33–36]. Prolonged administration of allopurinol frequently induces xanthine urolithiasis [37]. Thus, there is an urgent need for new, efficient, safe, and affordable drugs for the treatment of canine leishmaniasis.

Artemisinin and derivatives (ARTs) have demonstrated efficacy against protozoan parasites, such as *Plasmodium* [38] and *Perkinsus* species [39]. The antiparasitic activity of ARTs against other human and animal protozoans, namely, *Leishmania* spp., has scarcely been explored. Chollet et al. [40] reported on the activity of fluoroartemisinins against promastigote forms of *L*. *donovani* (at micromolar concentrations). Other compounds, such as artesunate, deoxygenated artesunate, dihydroartemisinin, and deoxygenated dihydroartemisinin, have shown in vitro activity against *L*. *infantum* life stage forms (promastigote and amastigote) [41]. In addition, ARTs showed efficacy for the treatment of murine visceral leishmaniasis [42–45].

In this study, we explored for the first time the efficacy of artesunate in the treatment of VL in *L*. *infantum* naturally infected dogs. Our study is an open-label simple randomized field trial includes two groups of sick dogs: i) control: dogs treated with the current chemotherapy (antimoniate of meglumine/ allopurinol); and ii) test, alternative chemotherapy: dogs treated with artesunate. Dogs were followed up for their clinical, parasitological and serological status.

## Materials and methods

### Ethics statement

The study protocol was also approved by the scientific college (Procès-Verbal du CSI N°6, 2018) at the Veterinary Science Institute, University Constantine 1, Algeria. To facilitate field work, collaborations were established with veterinary doctors and their assistants working in these establishments. All dog owners gave their verbal informed consent, and samples were collected by veterinarians. Risk assessment was also submitted to and approved by the decision board of the veterinary practitioners from the wilayas of northern Algeria affiliated with the Algerian Ministry of Agriculture and Rural Development (Directions des Services Vétérinaires). No infected untreated dogs, as control, were involved in this study for ethical reasons.

### Study design

The objective of the study is to test efficacity of artesunate in the treatment of CanL. We performed an open-label randomized, efficacy evidence clinical field trial conducted in the Kabylia, North of Algeria. We performed an imbalanced simple randomization. All animals in this study were exposed to the same epidemiological pressure and CanL prevalence recently observed in this area was 36% [19]. Client-owned dogs of any age, breed, or gender were enrolled, and 11 veterinary practitioners were responsible for the monitoring. The dogs were fully monitored, and treatment could be switched to antimoniate meglumine/allopurinol at the owner’s request if any clinical impairment was noticed. A total of 187 dogs were screened for compliance with the inclusion criteria. Among them, 42 sick adult dogs (30 males and 12 females), average age 3.8 years (min = 1, max = 11 years), weighing between 3–37 kg and of different breeds (8 German shepherd, 1 Belgian Malinois shepherd, 2 Sloughi, 5 pointer, 6 Braques, 1 Bagnole, 1 American Staffordshire terrier, 1 beagle and 17 mixed breeds) were selected for this study.

### Inclusion, exclusion and efficacy criteria

The main inclusion criteria were: i) dogs with at least two clinical manifestations related to CanL (Table 1) and ii) dogs with positive serology for *Leishmania* by immunochromatographic-based qualitative rapid WITNESS *Leishmania* test (Zoetis, France) and/or quantitative indirect immunofluorescence antibody test (IFAT≥100) or positive PCR result obtained from a blood sample.

**Table 1:** Manifestations related to CanL defined to evaluate the clinical scores of dogs.

*Symptoms*	*Number of signs*	*Clinical manifestations*
***General conditions***	5	Emaciation, anorexia, abbatement, anemia, hyperthermia
***Cutaneous signs***	6	Localized alopecia, pruritus, squamosis, hyperkeratosis, onychogriffosis, ulcers
***Visceral lesions***	4	Lymphadenopathy, splenomegaly, hepathomegaly, renal disorders
***Ophthalmic level***	4	Depilation around the eyes "in glasses", mucopurulent conjunctivitis, keratitis and/or uveitis
***Oral level***	1	Oral erosions
***Skeletal level***	3	Arthrites, synovitis, diffuse pain of the posterior train
***Others***	3	Epistaxis, chronic diarrhea, neurological signs

Each manifestation was assigned a severity score ranging from 0 to 3, where 0 = none, 1 = mild, 2 = moderate, and 3 = severe. Clinical scores varied [3–21]. Mortality related to CanL was assessed by the highest score (22).

The dogs with significant alterations in renal and/or hepatic function or with other clinically evident infectious diseases were excluded. The dogs were excluded if they had been vaccinated against or had been treated with leishmanicidal agents less than 3 months before preinclusion in the present study or with a leishmanistatic 1 month before preinclusion. Finally, no previous treatment with systemic antibiotics or antifungals within 7 days or with systemic long-acting corticosteroids within 1 month prior to preinclusion was allowed. Dogs were also free from other haemoprotozoan infections such as *Babesia* and *Trypanosoma* spp.. Pregnant and lactating dogs were excluded. Dogs could be withdrawn from the study at any time if they showed intolerance to the treatment or if requested by the owner.

The efficacy criteria were determined by reduction in clinical scores (i.e., the sum of clinical scores was lower than before treatment) [16,36,46], which was a decrease in parasite DNA and *Leishmania*-specific antibody titers. The score was defined according to the severity of each clinical sign, and the final value was obtained from the sum of all the values (Table 1). Quantitative parasite load was estimated through qPCR and antibody titers through quantitative IFAT.

### Experimental design

**Fig 1:**
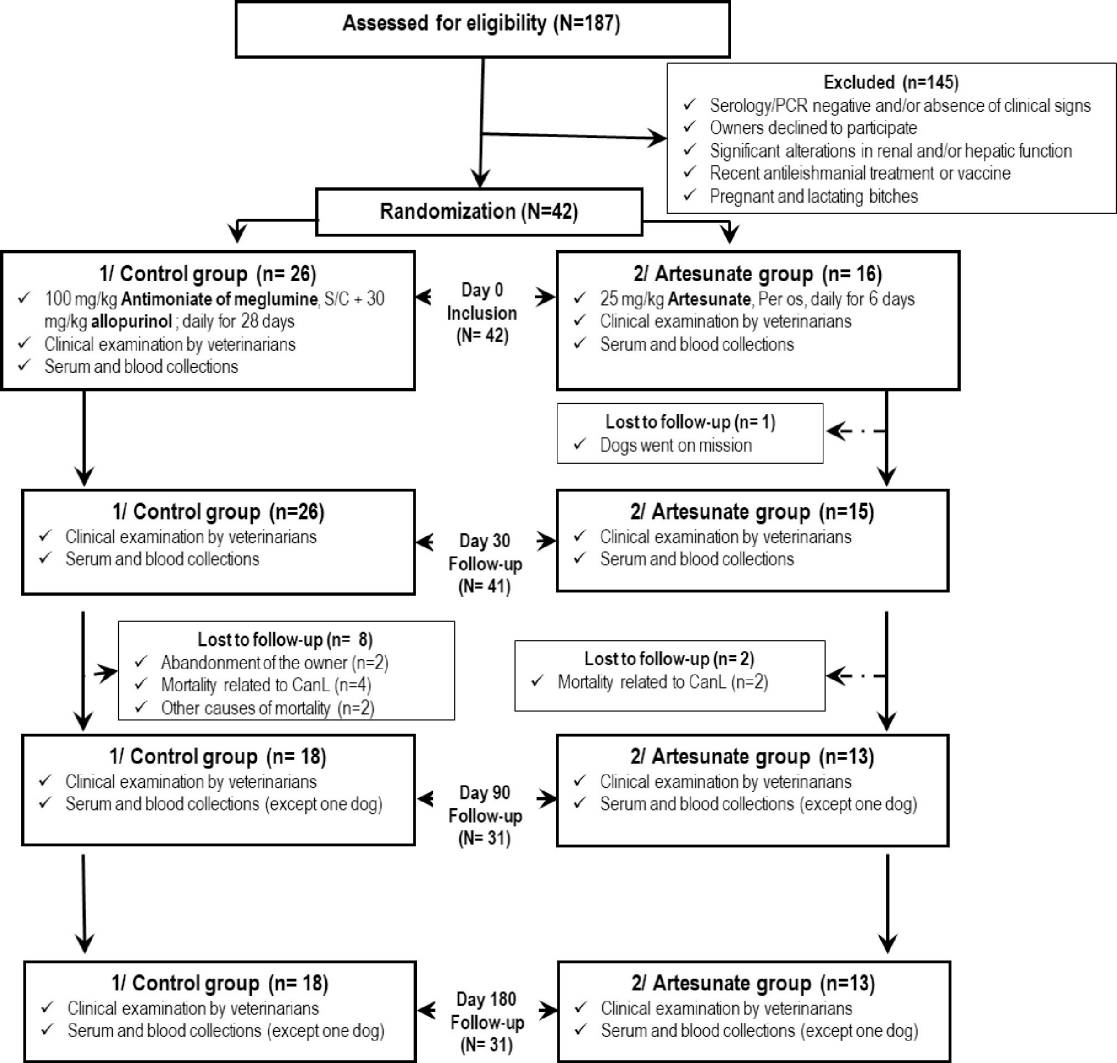
Consort flow diagram showing the therapeutic protocols and dogs with leishmaniasis in each stage of the study. Over all the 42 dogs included at D+0, one dog from the Group 2 was lost to follow-up before the D+30 time point. During the 6-month follow-up, four dogs from Group 1 were lost to follow-up two months after the beginning of treatment, and two of these were due to failure of clients to return for the final revisits on D+90 and D+180. Two others were because of death not related to CanL. In addition, six dogs died in the period between D+60 after the beginning of treatment and before the D+90 visit with increase in clinical signs related to CanL. Four of these were in Group 1, and two were in Group 2.

Selected dogs were randomly included in one of the two treatment arms (Table 2). Dogs (N = 26) in **Group 1** (positive control) received 100 mg/kg antimoniate of meglumine (Glucantime, Merial, France) daily for 28 days, subcutaneously, and 30 mg/kg allopurinol (Allopurinol Arraw 300 mg, France) daily for one month, orally. Dogs (N = 16) in **Group 2** (test group) received 25 mg/kg artesunate (Asu-Denk; Denk Pharma, Germany) daily for 6 days, orally. After each administration, the dogs were observed for 30 min to monitor for reactivity, vomiting and/or regurgitation to ensure complete absorption of the drug. The dogs were followed up for 180 days (Fig 1). To facilitate the trial, we performed an imbalanced simple randomization. This last was more balanced at the end of the trial and allowed environ 98% efficient as a balanced simple randomization.

**Table 2:** Characteristics of dogs assigned to each treatment arm and homogeneity analysis data, expressed as the mean ± standard deviation and frequencies (%).

Variable	Control treatment (Glucantime+ Allopurinol) (n = 26)	Test treatment (Artesunate) (n = 16)
**Age, year**	3.71 ± 2.11	3.94 ± 1.45
(min- max)	(1–11)	(2.5–8)
**Gender, male (%)**	17 (65.39)	13 (81.25)
**Breed**		
Breeder (%)	17 (65.39)	8 (50)
Mongrel (%)	9 (34.61)	8 (50)
**Clinical score:** points	7.46 ± 4.04	6.67 ± 2.30
(min- max)	(3–21)	(3–11)
**No. of positive by PCR (%)**	7 (26.92)	5 (31.25)
**qPCR-Parasitemia (Leish/mL)**	1108.72 ± 4154.5	78.04 ± 98.98
(min- max)	(0–21000)	(0–233)
No. Positive PCR (%*)	7 (26.92)	5 (31.25)
**Leishmania Antibody titers**	476.92 ± 448.38	390.62 ± (507.35)
(min- max)	(100–1600)	(50–1600)

%*: (Number of positive by PCR/Number of total positive) x 100.

#### Visit schedule and sample collection

The animals were checked on days D0, D+30, D+90 and D+180 after the treatment had started, and blood samples were collected at each time point. Clinical signs and lesions associated with leishmaniasis, infection status (parasite load-qPCR and antibody titers) were evaluated and monitored at these points.

#### Clinical evaluation

The efficacy of treatments was assessed at each time point based on the clinical response to treatment (evolution of clinical scores (CS) over time and the percentage reduction of the total clinical score). For each dog, the same veterinarian conducted all clinical evaluations to maintain consistency. Veterinarians complete a questionnaire, of the clinical signs that we can found, at each clinical examination (S1 Table). The CS were calculated according to the severity of clinical signs listed in Table 1, as reported previously [33,46]. Each manifestation was assigned a severity score ranging from 0 to 3, where 0 = none, 1 = mild, 2 = moderate, and 3 = severe (maximum total clinical score = 21). Mortality related to CanL was assessed by the highest score (22). The CS is the sum of scores of clinical signs. The reduction percentage was calculated according to the formula:

Percentage reduction of CS % = (CS at D+xx- CS at D+0)/CS at D+0) x 100.

CS: clinical score; D+xx: at Day xx post-treatment (D+30, D+90 or D+180), D+0: at the inclusion (day 0).

Clinical signs found during clinical examination, their scores, clinical scores and the reduction percentages of clinical signs, for each dog at each time-point, are presented in the S2 Table.

#### Parasite load-qPCR

Blood samples collected at each time point were analyzed for quantification of parasitic load by qPCR using primers and probe for detection and quantification of *L*. *infantum* DNA, targeting a conserved region of the kinetoplast minicircle DNA (kDNA) (several 1000-fold repeated sequence) [47]. Prior to DNA extraction, 200 μL of blood was digested with proteinase K and incubated at +56°C overnight. Extraction was performed using a commercial DNA extraction kit (QIAamp DNA Mini Kit, [Qiagen, Courtaboeuf, France]) and was performed on a BIOROBOT EZ1 (Qiagen, Qiagen, Courtaboeuf, France) per the manufacturer’s instructions. DNA was eluted in 200 μL of distilled water and stored at -20°C until analysis. The qPCR was prepared and performed as described in [6]. Briefly, 20 μL final volume containing 10 μL of Eurogentec Master Mix Roche (Eurogentec, Liège, Belgium), 0.5 mM of each primer and the probe, 0.5 μL UDG, 3 μL of distilled DNAse- and RNAse-free water, and 5 μL of the DNA sample was amplified in a CFX96 Real-Time system (BioRad Laboratories, Foster City, CA, USA) using the following thermal profile: one incubation step at 50°C for two minutes and an initial denaturation step at 95°C for three minutes, followed by 40 cycles of denaturation at 95°C for 15 seconds and annealing and extension at 60°C for 30 seconds. Quantification was based on a standard curve, which was an 8-fold serial dilution of 10^8^ copies of plasmid DNA/mL from the kDNA region, equivalent to 10000 parasites/mL, and 5 μL of serial dilutions ranging from 10000 to 0.001 parasites/mL was introduced into reaction tubes. The results were expressed as the number of *Leishmania* parasites present in 1 mL of blood, taking into account the volume (200 μL of blood) and the elution (200 μL) introduced during the extraction process. The percentage reduction in parasite load (PL%) was calculated according to the formula:

PL%=(PLatD+xx‐PLatD+0)/PLatD+0)x100.

PL: blood parasite load (Leish/mL); D+xx: at Day xx post-treatment (D+30, D+90 or D+180), D+0: at the inclusion day.

#### Serological monitoring

At day 0, the immunochromatographic-based qualitative rapid WITNESS *Leishmania* test (Zoetis, France) was performed according to the manufacturer’s instructions using one drop (10 μL) of whole blood from each dog directly after sampling. Quantitative IFAT for the titration of anti-*L*. *infantum-*specific immunoglobulins G (IgG) was performed on sera for monitoring as described previously [19]. Briefly, plate wells were coated with 20 μL of *L*. *infantum* commercial antigens (Zoetis, France). After dilution to 1:50, 20 μL of every serum dilution was applied per well, and plates were incubated for 30 min at 37°C. Plates were washed twice with PBS for 5 min and once with distilled water. Then, 20 μL of anti-dog IgG conjugated with fluorescein isothiocyanate (FITC) (Sigma-Aldrich, St Louis, MO, USA) were added into each well. The plates were incubated for 30 min at 37°C in the dark. After another washing step, drops of mounting medium were added to the cover slips, and reading was performed. To avoid observation errors, all samples were examined by two different investigators, and positive and negative controls were added to each plate. All samples negative at 1:50 were considered negative. Positive results were further investigated using a five-fold serial dilution IFAT at 1:100 to 1:1600. At the greatest dilution, the samples were classified as high positive for *L*. *infantum* (> 1:1600). In addition, in the manner as for the parasite load, we calculated the percentage reduction in antibody titers.

To evaluate efficacity of treatments, we performed comparison of averages CS, parasitemia and antibody titers taken at different time points for both groups, Group 1 alone, and Group 2 alone. Treatments were compared between us by comparison of percentage reduction of CS, parasite load and *Leishmania* antibodies at each time point of the follow-up.

### Data analysis

XLSTAT Addinsoft version 2018.7 was used for the statistical analysis. A descriptive analysis of the data was performed according to the nature of the variables for each follow-up visit and assigned treatment. The nonparametric Kruskal-Wallis/bilateral test was used for comparison of averages taken at evaluation time points for both groups, Group 1 alone, and Group 2 alone. Multiple pairwise comparisons following the Dunn/bilateral test procedure was used for the CS, parasitemia and antibody titers. Treatment effects were compared between the two groups by analysis of percentage reduction of CS, parasite load and antibody titers at each time point of the follow-up. The Mann-Whitney test was used to compare the efficacy of treatments using the difference in percentage reduction of CS, parasitemia and antibody titers between D0 and D+30 days, D0 and D+90 days, D0 and D+180 days. The significance level used for the tests was 5% (Bonferroni Correction level of significance was 0.0083).

## Results

### Included dogs and monitoring

No significant difference was found for age, gender, breed (mongrel or purebred), clinical status of dogs, and mean parasitemia and *Leishmania* antibody titers between the two groups at the inclusion day (P-value> 0.05) (Table 2).

The clinical examination allowed estimation and monitoring of the clinical outcome of the selected dogs (S2 Table). It revealed the presence of general signs in 92.85% (39/42), cutaneous in 83.33% (35/42), visceral in 38.1% (16/42), ophthalmic in 23.8% (10/42), and musculo-skeletal in 4.76% (2/42). One dog (2.3%) showed oral erosion, another presented epistaxis, and two dogs (4.76%) had neurological disorders.

Statistical analysis was performed on the number of dogs present at each time-point. Over all the 42 dogs included at D+0, one dog from the Group 2 was lost to follow-up before the D+30 time point. During the 6-month follow-up, four dogs from Group 1 were lost to follow-up two months after the beginning of treatment, and two of these were due to failure of clients to return for the final revisits on D+90 and D+180. Two others were because of death not related to CanL. In addition, six dogs died in the period between D+60 after the beginning of treatment and before the D+90 visit with increase in clinical signs related to CanL. Four of these were in Group 1, and two were in Group 2 (Fig 1).

### Clinical outcomes

**Fig 2:**
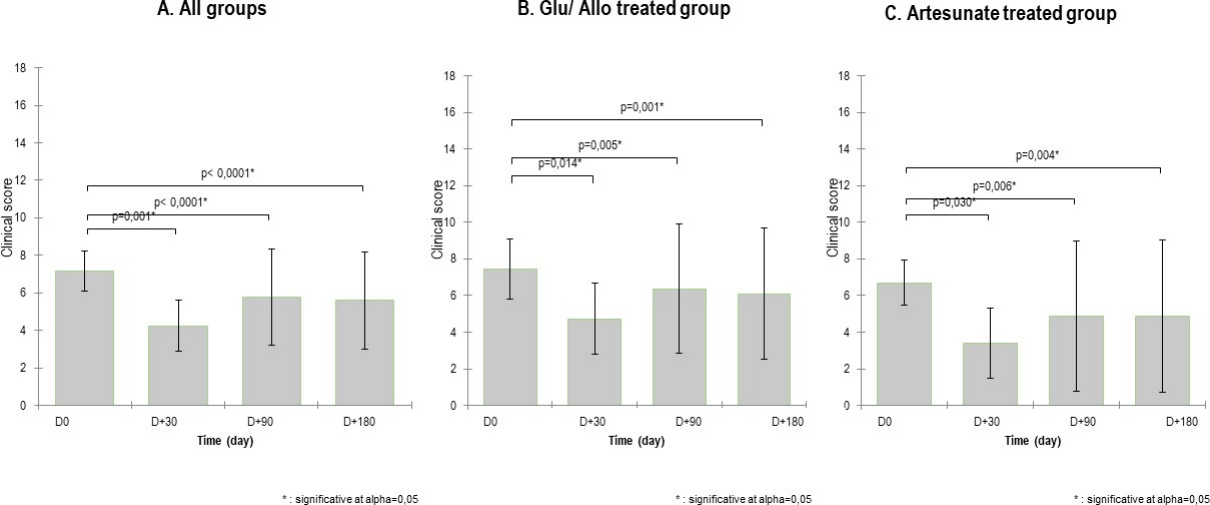
Time course of total clinical score (Kruskal-Wallis test). Clinical scores (mean ± SD) of the dogs (n = 42) were assessed by veterinarians at the time of scheduled visits at D0, D+30, D+90 and D+180. **A.** All treated groups. **B.** Glucantime/Allopurinol-treated group. **C.** Artesunate-treated group.

Compared to the initial clinical score, improvement in total clinical scores in both treatment groups was observed throughout the study. Given the mortality outcome of the higher score (22), mean clinical scores decreased over time from 7.16± 3.47 at D0 to 4.24± 4.37 at visit D+ 30, increased again slightly at D+ 90 (5.75± 8.25), and then decreased below the level at D0 and remained constant (5.60± 8.35) until D+ 180. The same clinical score kinetics were observed in the two groups, separately (Fig 2).

**Fig 3:**
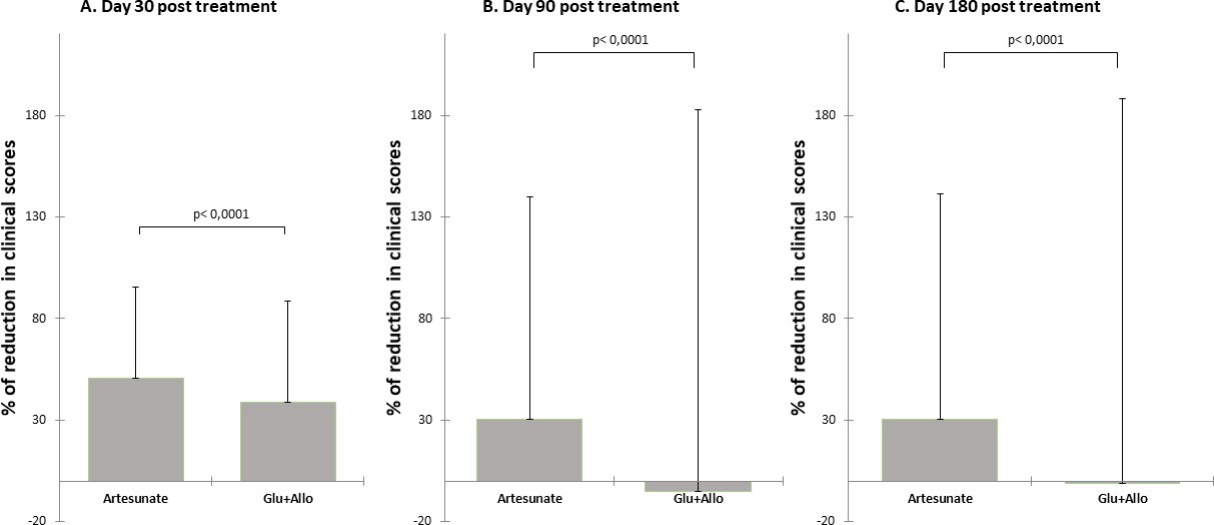
**Comparison of the mean percent reduction in clinical score between groups** (Mann-Whitney test); **A**. Day 30 posttreatment; **B**. Day 90 posttreatment; **C**. Day 180 posttreatment. Mortality was taken into consideration (mortality score = 22). The mean percent reduction in clinical score is higher in the artesunate-treated group than in the Glucantime/allopurinol-treated group at all time points.

Taking the CS at D0 and D+30 as the starting and end points, respectively, the mean percentage of reduction in clinical score was 43.3±47.8%. At each time point, D+30, D+90 and D+180, a statistically significant difference (p<0.0001) in mean percentage of reduction in clinical score was observed between the two groups. More improvement was noted in Group 2 than in Group 1 (Fig 3). At the level of individual response, approximately 26.66% (4/15) of Group 2 dogs complete recovered (CS of 0) *versus* 18.18% (4/22) in Group 1, while approximately 50% of the dogs showed partial amelioration at 6 months of follow-up in both groups with 54.54% (12/22) and 46.66% (7/15) in the control and test groups, respectively. At D+180, clinical worsening rates were similar in both groups at 27.27% (6/22), including 18.18% (4/22) mortality in the control group, *versus* 26.66% (4/15), including 13.33% (2/15) mortality, in the test group. Details on clinical changes over time for the dogs followed are described in Table 3.

**Table 3:** Individual clinical changes in dogs over time.

Groups	Visit	Complete amelioration (%)	Partial amelioration (%)	No change (%)	Aggravation (%)
**Artesunate treated dogs**	D+30 (n = 15)	-	12 (80)	-	3 (20)
	D+90 (n = 15)	3 (20)	8 (53.33)	1 (6.66)	3 (20)
	D+180 (n = 15)	4 (26.66)	7 (46.66)	-	4 (26.66)
**Glucantime/allopurinol treated dogs**	D+30 (n = 26)	2 (7.69)	17 (65.38)	1 (3.84)	6 (23.07)
	D+90 (n = 23)	3 (13.04)	13 (56.52)	1 (4.34)	6 (26.08)
	D+180 (n = 22)	4 (18.18)	12 (54.54)	-	6 (27.27)
**Total dogs**	D+30 (N = 41)	2 (4.87)	29 (70.73)	1 (2.44)	9 (21.91)
	D+90 (N = 38)	6 (15.79)	21 (55.26)	2 (5.26)	9 (23.68)
	D+180 (N = 37)	8 (21.62)	19 (51.35)	-	10 (27.02)

Mortality related to CanL had been included.

### Adverse events

No major side effects related to these treatments were reported in any dog. Both study compounds were well tolerated in all dogs, except for two dogs that had slight local pains and pruritus with 15 days of treatment with Glucantime/allopurinol.

### Quantitative real-time PCR kinetic parasitemia

At D0, 28.5% (12/42) dogs were positive by qPCR from a blood sample. Parasitic clearance was observed over time after treatment with artesunate, and all initially positive dogs (5/5) turned out to be *Leishmania*-negative. In contrast, 6/7 PCR-positive dogs treated with Glucantime/allopurinol turned out to be negative, and 1/7 dogs remained positive. In addition, two dogs treated with Glucantime/allopurinol were found to be positive, whereas they had been negative before. Individual evolution of parasitemia over the follow-up is shown in Table 4.

**Table 4:** Individual change in parasite load, number of parasites per mL of blood and percentage reduction over time.

Dog’s ID	Treatment	Parasitemia No. Leish/mL of blood (% of reduction)			
		D0	D+ 30	D+ 90	D+ 180
**13**	Glucantime/ Allopurinol	1500	288,7 (80,75)	30,28 (97,98)	357 (76,2)
**5**		0	0 (0,00)	176 (-100)*	177 (-100)*
**A11N12**		0	46,6 (-100) *	N/A*	N/A*
**S26**		2400	19,8 (99,18)	2 (99,92)	0 (100)
**S27**		3800	0 (100)	0 (100)	0 (100)
**S33**		21000	0 (100)	0 (100)	N/A
**S54**		7,7	0 (100)	0 (100)	N/A
**C4P2G2**		101	16,2 (83,96)	0 (100)	0 (100)
**C1G2P4**		18	0 (100)	0 (100)	0 (100)
**10**	Artesunate	6,8	0 (100)	0 (100)	0 (100)
**S50**		17,4	14 (19,54)	29 (-66,67)	0 (100)
**A9N9**		122	44 (63,93)	N/A	N/A
**T11C2P1**		233	1,7 (99,27)	0 (100)	0 (100)
**A8 N8**		11	0,17 (98,45)	0 (100)	0 (100)

All the other dogs were negative and remained negative during all of the follow-up.

N/A*: Mortality related to CanL

N/A: Lost to follow-up

### Serological status

**Fig 4:**
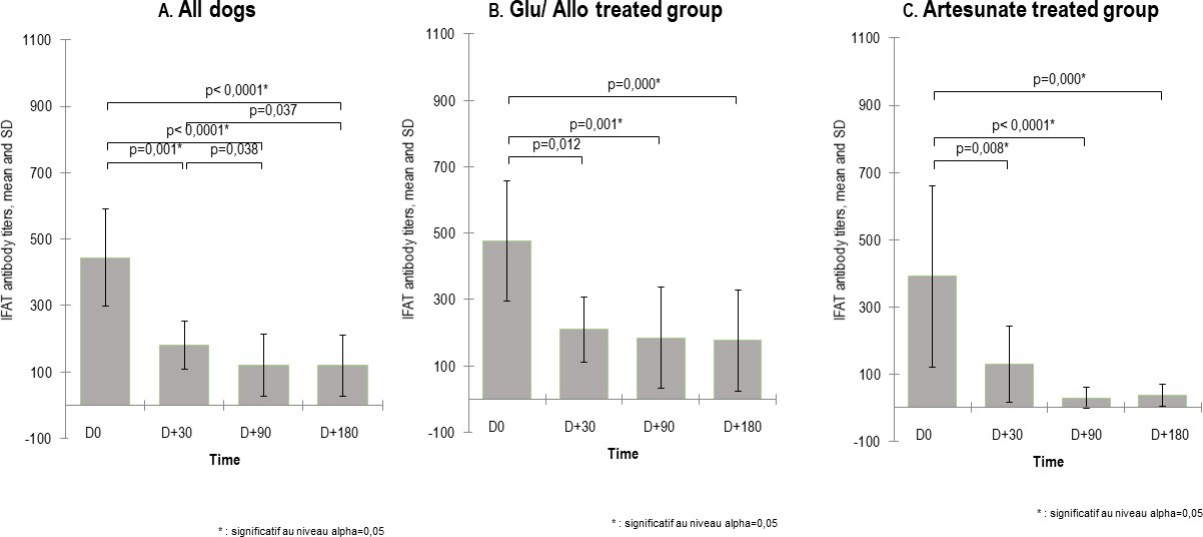
Serology monitoring. Antibody titers (mean ± SD) of the dogs (n = 42) were assessed by quantitative IFAT at the time of scheduled visits at D0, D+30, D+90 and D+180 (Kruskal-Wallis test/bilateral). **A.** All dogs. **B.** Glucantime/allopurinol group. **C.** Artesunate group.

Initially, all dogs were positive by both Witness test and IFAT, and no significant difference (P > 0.05) was observed between groups. The mean *Leishmania* antibody titers decreased over time (mean ± SD, 444.05 ± 467.50 at D0, 181.25 ± 234.71 at D+ 30, 120.69 ± 298.36 at D+ 90 and 118.97 ± 297.73 at D+ 180) after treatment (Fig 4). Compared to baseline values, significant reductions (P < 0.012, IFAT serology) were observed in both groups at D+30 days (mean ± SD, artesunate 128.57 ± 212.78; Glucantime/allopurinol 209.61 ± 244.96). These levels continued to decrease (mean ± SD, artesunate 37.50 ± 60.77; Glucantime/allopurinol 176.471 ± 379.604) at D+ 180 (Fig 3). When groups were compared, the artesunate group showed a higher percent reduction in antibody titers at the end of the study (P <0.0001) (S1 Fig). At the individual level, 78.57% (11/14) of dogs treated with artesunate showed a decrease in antibody titers at D+30, and 50% (7/14) of them became absolutely seronegative. In contrast, in the Glucantime/allopurinol group, 57.7% (15/26) showed a decrease in serology at D+30, and only 15.38% (4/26) became seronegative. At the end of the follow-up, 41.37% (12/29) of dogs became seronegative, including 29.41% (5/17) in Group 1 *versus* 58.33% (7/12) in Group 2 (P-value, Z test/bilateral = 0.227).

## Discussion

Today, there is lack of an effective and safe therapy against canine visceral leishmaniasis. New drugs, delivery systems and treatment strategies are necessary to achieve a cure in infected dogs [48]. This is important not only for dogs but also as a part of the fight against human leishmaniasis (reservoir sanction). Here, we compared the efficacy of artesunate as an alternative chemotherapy and Glucantime/allopurinol, which is currently the most effective treatment against CanL [49]. The treatment responses were determined using a comprehensive clinical score, which was based on the main clinical signs associated with CanL, i.e., parasite load evolution using qPCR and serological monitoring by measurement of antibody titers through quantitative IFAT [25,50,51]. This is the first report on the efficacy of artesunate (an artemisinin derivative) in the treatment of CanL in dogs under field conditions.

Both treatments led to clinical improvement and reduced parasite burden and *Leishmania* antibody titers, but a greater improvement in clinical signs was observed with artesunate. Artemisinin and its derivatives have proven therapeutic potential against malaria and have also demonstrated effectiveness in experimental models of leishmaniasis [42,52]. Artemisinin was active against six strains of *Leishmania* responsible for diverse forms of leishmaniasis [42]. In this study, artesunate was well tolerated without any adverse effects. In one study, artesunate was used in dogs as an anti-cancer treatment; dogs exhibited no adverse effects at all [53]. Two dogs in the control group showed local pain at the site of injection. Treatment in the control group was by injection+ oral administration, while artesunate was given by oral administration. Side effects after treatment with meglumine antimoniate/allopurinol that have been previously demonstrated were mainly as follows: local pain, pancreatitis, panniculitis and nephrotoxicity for meglumine antimoniate, and xanthinuria, urolithiasis and renal mineralization for allopurinol [31,33], where it has been observed that 16% of dogs developed adverse events after treatment with Glucantime.

A rapid improvement in general conditions was observed a few days after treatment with artesunate. At the D+30 visit, 80% of dogs in the test group *versus* 73% in the control group showed clinical amelioration. Clinical improvement occurred rapidly in dogs 30 days after meglumine antimoniate/allopurinol therapy in several studies [27,49,54–56]. Treatments were successful in almost ¾ of cases and failed in almost ¼ of the cases after six months follow-up in both groups. Worsening was observed in 6 dogs *versus* 4 dogs (including the deaths of 4 dogs *versus* 2 dogs) in the control *versus* test groups. In one study, 10 months after the end of combined meglumine and allopurinol treatment in 6 dogs, 4 dogs had no clinical signs, 2 had relapsed, and the amastigote forms of *Leishmania* were present in the spleen of 5 dogs [49]. In addition, we estimate that Glucantime/allopurinol treatment is at least 10-fold more expensive than artesunate. In this study, artesunate showed a greater reduction in clinical scores than Glucantime/allopurinol. Compared to the current treatment, which is very burdensome and complicated in practice, artesunate treatment could be a good alternative chemotherapy, especially because it is cheap and easy to use (*per os*) for a short period (6 days of treatment in the present study) [31]. Furthermore, its efficacy can be improved by adjusting the dose or period of treatment, and further investigations can be used to determine the best regimen.

The blood PCR technique used had less diagnostic value than quantitative serology, and only 12/42 of sick seropositive dogs were found to be PCR-positive. This finding is in agreement with other authors [26,57] the fact that it is well known that blood parasitemia might be intermittent [30,58,59], and the blood *Leishmania* parasite load is much lower than the load found in other tissues, such as bone marrow in dogs with clinical leishmaniasis [57,60,61].

Parasitemia decreased continuously after treatment with artesunate until its total disappearance (100% parasite clearance, 180 days post-treatment), suggesting a leishmanicidal activity for this compound. The leishmanicidal activity of artemisinin and derivatives against *Leishmania* spp., causative agents of New World and Old World leishmaniases (including *L*. *infantum*), have been demonstrated in vitro [41] and in a mouse experimental model [42]. In this study, Glucantime/allopurinol greatly reduced the parasite load at the end of treatment (4/7 initially positive dogs became PCR negative at D+ 30). A significant decline in the blood parasite load during the first 30 days using this combination therapy has been reported [26,62]. In addition, all PCR-negative dogs remained negative after treatment with artesunate; in contrast, two dogs in the control group became PCR-positive with worsening of clinical signs at D+ 90 follow-up. Failures of the combination Glucantime/allopurinol therapy have been reported, mainly due to the drug-resistance of *Leishmania*, but time to relapse in treated dogs has not been documented [31]. In our case, relapse started the second month posttreatment.

The present study showed that 30 days following the initiation of treatment, there was an important significant decline (2.28- *versus* 3.04-fold, for control *versus* test group) in *L*. *infantum*-specific antibody titers corresponding with clinical improvement as has been reported in previous studies [26,63]. Antibody levels continued to decrease more in the test group, where 58% of cases became seronegative 6 months posttreatment (Fig 3). This indicates a regression in antigenic stimulation [48,64]. A positive association has been found between antibody levels and parasitic dissemination to different tissues [65,66]; therefore, it might be hypothesized that the decrease in antibody levels implies that there is no parasitic dissemination. Compared to other studies, the antibody level continued to decrease progressively but slowly during the treatment period [55,67,68]. As has been shown in previous studies, when compared to the baselines, only a minority of dogs became seronegative during the first year of treatment, but almost all of them reached much lower antibody levels [69,70]. Our finding showed that artesunate induces a rapid and greater reduction in antibody levels than the combination therapy Glucantime/allopurinol. Moreover, compared to day 0, clinical worsening and death in all cases were associated with increasing or at least stable antibody levels without any serological resolve. Therefore, we corroborate that serological monitoring is very useful for the detection of clinical relapse and prognosis after treatment since it correlates with an increase in antibody titers [25,26,29,55].

### Study limitations

The study is innovative because it uses an antiprotozoal drug that has never been used to treat CanL. However, it presents certain limits that were required to us by the difficulties encountered in the field: i) the number of dogs followed remains insufficient (42 dogs at the beginning and only 31 at the end of the follow-up). ii) We carried out an imbalanced simple randomization, it would be better to do a balanced randomization with equal number of dogs in each group. Another weakness of this study is the use of blood for parasite load since it is known that *L*. *infantum* is a parasite of mononuclear phagocyte system cells and it has a tropism for bone marrow, lymph node and other internal organs like spleen and kidney. The number of parasites in blood is low and it was not possible to carry out other sampling type, so most of dog owners refused it. For this reason, we do not have many positive cases in blood PCR. Another point, biochemical analysis, especially creatinine and urea dosage, haven’t been performed. Ultimately, co-infection can influence the clinical course of CanL. We looked at co-infections with other protozooses, such as *Babesia* spp. and *Trypanosoma* spp. Dogs in this trial were not co-infected at the time of inclusion. Unfortunately, we did not look at *Ehrlichia canis* infection, which also may influence the clinical course of CanL. We would like to see these limitations taken into account in future studies to better confirm the results on the efficacy of artesunate in the treatment of CanL.

## Conclusions

These findings indicate that artesunate as well as combination meglumine antimoniate/allopurinol are effective treatments against canine visceral leishmaniasis. Artesunate was safer and more effective in controlling and reducing the clinical signs of leishmaniasis, parasite load and antibody titers than meglumine antimoniate/allopurinol. Furthermore, it is cheaper and easier to administrate (orally) with a short treatment period.

Now, it is important to obtain more data on oral artesunate treatment in other trials with a larger number of dogs and long-term follow-up. Further clinical trials are needed to confirm our results, also to determine if there are relapses after treatment and if dogs remain infective to sandflies, to define the ideal therapeutic dosage and duration of treatment. Finally, because the dog is an ideal model for human visceral leishmaniasis (VL) studies, the efficacy of artesunate in the treatment of VL should be evaluated.

## Supporting information

S1 Fig Comparison of mean percent reduction in IFAT antibody titers in dogs with leishmaniasis treated with artesunate or Glucantime/allopurinol. Data are reported as the mean ± SD, artesunate *versus* Glucantime/allopurinol (Mann-Whitney test) 30-, 90- and 180-days posttreatment.
(TIF)

S1 Table Questionnaire completed by veterinarians about dog information and clinical manifestations. (DOCX)

S2 Table Individual clinical signs and calculation of clinical scores (CS) and percentage reduction of CS at each time-point. (XLSX)

## References

[1] Caixeta I, Helena L, Coura-vital W, Macedo G, Magalhães C, Almeida T, et al Effectiveness of the Brazilian Visceral Leishmaniasis Surveillance and Control Programme in reducing the prevalence and incidence of Leishmania infantum infection. Parasites & Vectors; 2018; 1–12. 30419944 10.1186/s13071-018-3166-0 PMC6233359

[2] Dantas-Torres F, Brandão-Filho SP. Visceral leishmaniasis in Brazil: revisiting paradigms of epidemiology and control. Rev Inst Med Trop Sao Paulo. 2006;48(3):151–156. 10.1590/s0036-46652006000300007 16847505

[3] Desjeux P. Leishmaniasis: current situation and new perspectives. 2004;27: 305–318. 10.1016/j.cimid.2004.03.004 15225981

[4] Salomón OD, Feliciangeli MD, Quintana MG, Martins M, Rangel EF. *Lutzomyia longipalpis* urbanisation and control. 2015;110: 831–846. 10.1590/0074-02760150207 26517497 PMC4660613

[5] Romero GAS, Boelaert M. Control of Visceral Leishmaniasis in Latin America—A Systematic Review. *Plos Neg Trop Dis*. 2010;4 10.1371/journal.pntd.0000584 20098726 PMC2808217

[6] Medkour H, Davoust B, Dulieu F, Maurizi L, Lamour T, Marié J-L, et al Potential animal reservoirs (dogs and bats) of human visceral leishmaniasis due to *Leishmania infantum* in French Guiana. PLoS Negl Trop Dis. 2019;13: e0007456 10.1371/journal.pntd.0007456 31216270 PMC6602241

[7] Courtenay O, Quinnell RJ, Garcez LM, Dye C. Low infectiousness of a wildlife host of *Leishmania infantum*: the crab-eating fox is not important for transmission. Parasitology. 2002;125(Pt 5):407–414. 10.1017/s0031182002002238 12458824

[8] Alvar J, Vélez ID, Bern C, Herrero M, Desjeux P, Cano J, et al Leishmaniasis worldwide and global estimates of its incidence. PLoS One. 2012;7 10.1371/journal.pone.0035671 22693548 PMC3365071

[9] Ready PD. Epidemiology of visceral leishmaniasis. Clin Epidemiol. 2014;6: 147–154. 10.2147/CLEP.S44267 24833919 PMC4014360

[10] Freitas-junior LH, Chatelain E, Andrade H, Siqueira-neto JL. International Journal for Parasitology: Drugs and Drug Resistance Visceral leishmaniasis treatment: What do we have, what do we need and how to deliver it ? Int J Parasitol Drugs Drug Resist. Australian Society for Parasitology; 2012;2: 11–19. 10.1016/j.ijpddr.2012.01.003 24533267 PMC3862432

[11] Baneth G, Aroch I. Canine leishmaniasis: A diagnostic and clinical challenge. Vet J. 2008;175: 14–15. 10.1016/j.tvjl.2006.11.011 17215150

[12] Ribeiro RR, Suzan M, Michalick M, Da Silva ME, Peixoto CC, Santos D, et al Canine Leishmaniasis: An Overview of the Current Status and Strategies for Control. Biomed Res Int. 2018;2018 10.1155/2018/3296893 29789784 PMC5896350

[13] Moreno J, Alvar J. Canine leishmaniasis: Epidemiological risk and the experimental model. Trends Parasitol. 2002;18: 399–405. 10.1016/s1471-4922(02)02347-4 12377257

[14] Leontides LS, Saridomichelakis MN, Billinis C, Kontos V, Koutinas AF, Galatos AD, et al A cross-sectional study of *Leishmania* spp. infection in clinically healthy dogs with polymerase chain reaction and serology in Greece. 2002;109: 19–27. 10.1016/s0304-4017(02)00201-7 12383622

[15] Miró G, López-Vélez R. Clinical management of canine leishmaniosis versus human leishmaniasis due to *Leishmania infantum*: Putting “One Health” principles into practice. Vet Parasitol. Elsevier; 2018;254: 151–159. 10.1016/j.vetpar.2018.03.002 29657002

[16] Nogueira S, Avino VC, Galvis-ovallos F, Pereira-chioccola VL, Antonio M, Moreira B, et al Use of miltefosine to treat canine visceral leishmaniasis caused by *Leishmania infantum* in Brazil. Parasites & Vectors; 2019;0: 1–11. 10.1186/s13071-019-3323-0 PMC6368741 30736866

[17] Dujardin JC, Campino L, Cañavate C, Dedet JP, Gradoni L, Soteriadou K, et al Spread of vector-borne diseases and neglect of leishmaniasis, Europe. Emerg Infect Dis. 2008;14: 1013–1018. 10.3201/eid1407.071589 18598618 PMC2600355

[18] Pineda C, Aguilera-Tejero E, Morales MC, Belinchon-Lorenzo S, Gomez-Nieto LC, Garcia P, Martinez-Moreno JM, Rodriguez-Ortiz ME, Lopez I. Treatment of canine leishmaniasis with marbofloxacin in dogs with renal disease. PLoS One. 2017 10 5;12(10):e0185981 10.1371/journal.pone.0185981 28982165 PMC5641981

[19] Medkour H, Laidoudi Y, Lafri I, Davoust B, Mekroud A, Bitam I, et al Canine vector-borne protozoa: Molecular and serological investigation for *Leishmania* spp., *Trypanosoma* spp., *Babesia* spp., and *Hepatozoon* spp. in dogs from Northern Algeria. Vet Parasitol Reg Stud Reports. Elsevier; 2020;19: 100353 10.1016/j.vprsr.2019.100353 32057382

[20] Adel A, Abatih E, Speybroeck N, Soukehal A, Bouguedour R, Boughalem K, et al Estimation of canine *Leishmania* infection prevalence in six cities of the Algerian littoral zone using a Bayesian approach. PLoS One; 2015;10: e0117313–e0117313. 10.1371/journal.pone.0117313 25793942 PMC4368835

[21] Zoghlami Z, Chouihi E, Barhoumi W, Dachraoui K, Massoudi N, Helel K Ben, et al Interaction between canine and human visceral leishmaniases in a holoendemic focus of Central Tunisia. Acta Trop. 2014;139: 32–38. 10.1016/j.actatropica.2014.06.012 25004438

[22] Gharbi M, Jaouadi K, Mezghani D, Darghouth A. Symptoms of Canine *Babesia* spp., and *Hepatozoon* spp in Tunisian Dogs. 2018; 51–55. 10.3166/bspe-2018-0017 30763509

[23] Boussaa S, Kasbari M, Mzabi A El, Boumezzough A. Epidemiological Investigation of Canine Leishmaniasis in Southern Morocco. Advances in Epidemiology. 2014; 104697 10.1155/2014/104697

[24] Bessad A, Mouloua K, Kherrachi I, Benbetka S, Benikhlef R, Mezai G, et al *Leishmania infantum* MON-1 isolé d’un chacal doré (*Canis aureus*) en Grande Kabylie (Algérie). Bull la Soc Pathol Exot. 2012;105: 5–7. 10.1007/s13149-011-0182-4 21874583

[25] Cardoso L. LeishVet guidelines for the practical management of canine leishmaniosis. Parasites & Vectors. 2011; 1–16. 10.1186/1756-3305-4-86 21599936 PMC3125381

[26] Solano-Gallego L, Di Filippo L, Ordeix L, Planellas M, Roura X, Altet L, et al Early reduction of *Leishmania infantum*-specific antibodies and blood parasitemia during treatment in dogs with moderate or severe disease. Parasites & Vectors; 2016;9: 1–9. 10.1186/s13071-016-1519-0 27160317 PMC4862152

[27] Oliva G, Cruz I, Veterinaria M, Ii F, Animal P. Multicentric, controlled clinical study to evaluate effectiveness and safety of miltefosine and allopurinol for canine leishmaniosis. 2009; 397–404. 10.1111/j.1365-3164.2009.00824.x 20178476

[28] Committee WHOE. Control of the leishmaniases. 2010; 22–26.

[29] Cardoso L, Pennisi MG, Solano-gallego L, Koutinas A, Miro G, Ferrer L, et al Veterinary Parasitology Directions for the diagnosis, clinical staging, treatment and prevention of canine leishmaniosis. 2009;165: 1–18. 10.1016/j.vetpar.2009.05.022 19559536

[30] Travi BL, Cordeiro-da-Silva A, Dantas-Torres F, Miró G. Canine visceral leishmaniasis: Diagnosis and management of the reservoir living among us. PLoS Negl Trop Dis. 2018;12(1):e0006082 Published 2018 Jan 11. 10.1371/journal.pntd.0006082 29324838 PMC5764232

[31] Miró G, Petersen C, Cardoso L, Bourdeau P, Baneth G, Solano-gallego L, et al Novel Areas for Prevention and Control of Canine Leishmaniosis. Trends Parasitol. Elsevier Ltd; 2017;xx: 1–13. 10.1016/j.pt.2017.05.005 28601528

[32] Noli C, Auxilia ST. Treatment of canine Old World visceral leishmaniasis: a systematic review. Vet Dermatol. 2005;16(4):213–232. 10.1111/j.1365-3164.2005.00460.x 16101793

[33] Mateo M, Maynard L, Vischer C, Bianciardi P, Miró G. Comparative study on the short term efficacy and adverse effects of miltefosine and meglumine antimoniate in dogs with natural leishmaniosis. 2009; 155–162. 10.1007/s00436-009-1375-3 19238439

[34] Bianciardi P, Brovida C, Valente M, Aresu L, Cavicchioli L, Vischer C, et al Toxicologic Pathology. 2009; 10.1177/0192623309344088 19690151

[35] Manna L, Vitale F, Reale S, Picillo E, Neglia G, Vescio F, et al Study of efficacy of miltefosine and allopurinol in dogs with leishmaniosis. Vet J. Elsevier Ltd; 2009;182: 441–445. 10.1016/j.tvjl.2008.08.009 18818112

[36] Woerly V, Maynard L, Sanquer A, Eun H. Clinical efficacy and tolerance of miltefosine in the treatment of canine leishmaniosis. 2009; 463–469. 10.1007/s00436-009-1404-2 19322588

[37] Saridomichelakis MN, Savvas I, Leondides L. Effects of Allopurinol Treatment on the Progression of Chronic Nephritis in Canine Leishmaniosis (Leishmania infantum). 2006; 228–233. 10.1892/0891-6640(2006)20[228:eoatot]2.0.co;2 16594576

[38] Loo CS, Lam NS, Yu D, Su XZ, Lu F. Artemisinin and its derivatives in treating protozoan infections beyond malaria. Pharmacol Res. 2017;117:192–217. 10.1016/j.phrs.2016.11.012 27867026 PMC5316320

[39] Araujo NCP, Afonso R, Bringela A, Cancela ML, Cristiano MLS, Leite RB. Parasitology International Peroxides with antiplasmodial activity inhibit proliferation of *Perkinsus olseni*, the causative agent of Perkinsosis in bivalves. Parasitol Int.; 2013;62: 575–582. 10.1016/j.parint.2013.06.010 23831484

[40] Bories C, Chollet C. In vitro antileishmanial activity of fluoro-artemisinin derivatives against *Leishmania donovani*. 2008;62: 462–465. 10.1016/j.biopha.2008.04.003 18538529

[41] Cortes S, Albuquerque A, Cabral LIL, Lopes L, Campino L, Cristiano LS. In Vitro Susceptibility of *Leishmania infantum* to Artemisinin Derivatives and Selected Trioxolanes. 2015;59: 5032–5035. 10.1128/AAC.00298-15 26014947 PMC4505222

[42] Sen R, Ganguly S, Saha P, Chatterjee M. Efficacy of artemisinin in experimental visceral leishmaniasis. Int J Antimicrob Agents. Elsevier B.V.; 2010;36: 43–49. 10.1016/j.ijantimicag.2010.03.008 20403680

[43] Want MY, Islamuddin M, Chouhan G, Ozbak HA, Hemeg HA, Dasgupta AK, et al Therapeutic efficacy of artemisinin-loaded nanoparticles in experimental visceral leishmaniasis. Colloids Surfaces B Biointerfaces. Elsevier B.V.; 2015;130: 215–221. 10.1016/j.colsurfb.2015.04.013 25936561

[44] Islamuddin M, Chouhan G, Farooque A, Dwarakanath BS, Sahal D, Afrin F. Th1-Biased Immunomodulation and Therapeutic Potential of *Artemisia annua* in Murine Visceral Leishmaniasis. PLoS Negl Trop Dis. 2015;9 10.1371/journal.pntd.0003321 25568967 PMC4287499

[45] Want MY, Islammudin M, Chouhan G, Ozbak HA, Hemeg HA, Chattopadhyay AP, et al Nanoliposomal artemisinin for the treatment of murine visceral leishmaniasis. Int J Nanomedicine. 2017;12: 2189–2204. 10.2147/IJN.S106548 28356736 PMC5367595

[46] Manna L, Reale S, Vitale F, Elio A. Research in Veterinary Science Evidence for a relationship between *Leishmania* load and clinical manifestations. Res Vet Sci. Elsevier Ltd; 2009;87: 76–78. 10.1016/j.rvsc.2008.12.009 19178919

[47] Mary C, Faraut F, Lascombe L, Dumon H. Quantification of *Leishmania infantum* DNA by a Real-Time PCR Assay with High Sensitivity. J Clin Microbiol. 2004;42: 5249–5255. 10.1128/JCM.42.11.5249-5255.2004 15528722 PMC525214

[48] Baneth G, Shaw SE. Chemotherapy of canine leishmaniosis. Vet Parasitol. 2002;106(4):315–324. 10.1016/s0304-4017(02)00115-2 12079737

[49] Manna L, Corso R, Galiero G, Cerrone A, Muzj P, Gravino AE. Long-term follow-up of dogs with leishmaniosis treated with meglumine antimoniate plus allopurinol versus miltefosine plus allopurinol. Parasites & Vectors; 2015; 1–9. 10.1186/s13071-015-0896-0 26017164 PMC4458061

[50] Roura X, Fondati A, Lubas G, Gradoni L, Maroli M, Oliva G, et al Prognosis and monitoring of leishmaniasis in dogs: A working group report. Vet J. Elsevier Ltd; 2013;198: 43–47. 10.1016/j.tvjl.2013.04.001 23680263

[51] Segarra S, Miró G, Montoya A, Pardo-marín L, Boqué N, Ferrer L, et al Randomized, allopurinol-controlled trial of the effects of dietary nucleotides and active hexose correlated compound in the treatment of canine leishmaniosis. Vet Parasitol. Elsevier; 2017;239: 50–56. 10.1016/j.vetpar.2017.04.014 28495197

[52] Yang DM, Liew FY. Effects of qinghaosu (artemisinin) and its derivatives on experimental cutaneous leishmaniasis. Parasitology. 1993;106 (Pt 1):7–11. 10.1017/s0031182000074758 8479804

[53] Rutteman GR, Erich SA, Mol JA, et al Safety and efficacy field study of artesunate for dogs with non-resectable tumours. Anticancer Res. 2013;33(5):1819–1827. 23645726

[54] Denerolle P, Bourdoiseau G. Combination allopurinol and antimony treatment versus antimony alone and allopurinol alone in the treatment of canine leishmaniasis (96 cases). J Vet Intern Med. 1999;13(5):413–415. 10.1892/0891-6640(1999)013&lt;0413:caaatv&gt;2.3.co;2 10499722

[55] Torres M, Bardagí M, Roura X, Zanna G, Ravera I, Ferrer L. Long term follow-up of dogs diagnosed with leishmaniosis (clinical stage II) and treated with meglumine antimoniate and allopurinol. Vet J. Elsevier Ltd; 2011;188: 346–351. 10.1016/j.tvjl.2010.05.025 20594876

[56] Alvar J, Molina R, San Andrés M, et al Canine leishmaniasis: clinical, parasitological and entomological follow-up after chemotherapy. Ann Trop Med Parasitol. 1994;88(4):371–378. 10.1080/00034983.1994.11812879 7979624

[57] Rodríguez-cortés A, Ojeda A, Francino O, López-fuertes L, Timón M, Alberola J. *Leishmania* Infection: Laboratory Diagnosing in the Absence of a “Gold Standard.” 2010;82: 251–256. 10.4269/ajtmh.2010.09-0366 20134001 PMC2813166

[58] Manna L, Gravino E, Picillo E, Decaro N, Buonavoglia C. Leishmania DNA Quantification by Real-time PCR in Naturally Infected Dogs Treated with Miltefosine. 2008;360: 358–360. 10.1196/annals.1428.018 19120249

[59] Paltrinieri S, Gradoni L, Roura X, Zatelli A, Zini E. Laboratory tests for diagnosing and monitoring canine leishmaniasis. Vet Clin Pathol. 2016;45: 552–578. 10.1111/vcp.12413 27805725

[60] Solano-gallego L, Rodriguez-cortes A, Trotta M, Zampieron C, Razia L, Furlanello T, et al Detection of *Leishmania infantum* DNA by fret-based real-time PCR in urine from dogs with natural clinical leishmaniosis. Elsevier B.V.; 2007;147: 315–319. 10.1016/j.vetpar.2007.04.013 17532143

[61] Maia C, Ramada J, Cristóvão JM, Gonçalves L, Campino L. Diagnosis of canine leishmaniasis: Conventional and molecular techniques using different tissues. Vet J. 2009;179: 142–144. 10.1016/j.tvjl.2007.08.009 17936654

[62] Martínez V, Quilez J, Sanchez A, Roura X, Francino O, Altet L. Canine leishmaniasis: The key points for qPCR result interpretation. Parasites and Vectors. 2011;4: 1–5. 10.1186/1756-3305-4-1 21489253 PMC3086858

[63] Oliva BYG, Gradon LI, Cortese L, Ella PC, Medica C, Ii ÁF, et al Comparative efficacy of meglumine antimoniate and aminosidine sulphate, alone or in combination, in canine leishmaniasis. Annals of Tropical Medicine and Parasitology, Volume 92, Number 2, 1 3 1998, pp. 165–171(7). 10.1080/00034989860003 9625912

[64] Altet L, Solano-gallego L, Sa E, Timo M, Francino O, Alberola J. A long term experimental study of canine visceral leishmaniasis. 2007;37: 683–693. 10.1016/j.ijpara.2006.11.007 17239885

[65] Martínez-Moreno A, Moreno T, Martínez-Moreno FJ, Acosta I, Hernández S. Humoral and cell-mediated immunity in natural and experimental canine leishmaniasis. Vet Immunol Immunopathol. 1995;48(3–4):209–220. 10.1016/0165-2427(95)05434-8 8578681

[66] Solano-gallego L, Morell P, Arboix M, Alberola J, Farmacologia D De. Prevalence of Leishmania infantum Infection in Dogs Living in an Area of Canine Leishmaniasis Endemicity Using PCR on Several Tissues and Serology. 2006;39: 560–563. 10.1128/JCM.39.2.560 PMC87775 11158106

[67] Solano-gallego L, Riera C, Roura X, Iniesta L, Enric J. *Leishmania infantum* -specific IgG, IgG1 and IgG2 antibody responses in healthy and ill dogs from endemic areas Evolution in the course of infection and after treatment. 2001;96: 265–276. 10.1016/s0304-4017(00)00446-5 11267753

[68] Pasa S, Ozensoy S, Voyvoda H, Ozbel Y. Clinical and serological follow-up in dogs with visceral leishmaniosis treated with allopurinol and sodium stibogluconate. 2005;128: 243–249. 10.1016/j.vetpar.2004.12.002 15740861

[69] Vercammen F, DeDeken R. Antibody kinetics during allopurinol treatment in canine leishmaniasis. Vet Rec. 1996;139(11):264 10.1136/vr.139.11.264-a 8888563

[70] Rodríguez A, Solano-Gallego L, Ojeda A. Dynamics of *Leishmania*-specific immunoglobulin isotypes in dogs with clinical leishmaniasis before and after treatment. J Vet Intern Med. 2006;20(3):495–498. 10.1892/0891-6640(2006)20[495:doliii]2.0.co;2 16734080

