# Supplementary figures and images for: Potential of Artesunate in the treatment of visceral leishmaniasis in dogs naturally infected by Leishmania infantum: Efficacy evidence from a randomized field trial
Source: PLoS Negl Trop Dis. 2020 Dec 18;14(12):e0008947. doi: 10.1371/journal.pntd.0008947 (PMC7781483; doi:10.1371/journal.pntd.0008947)

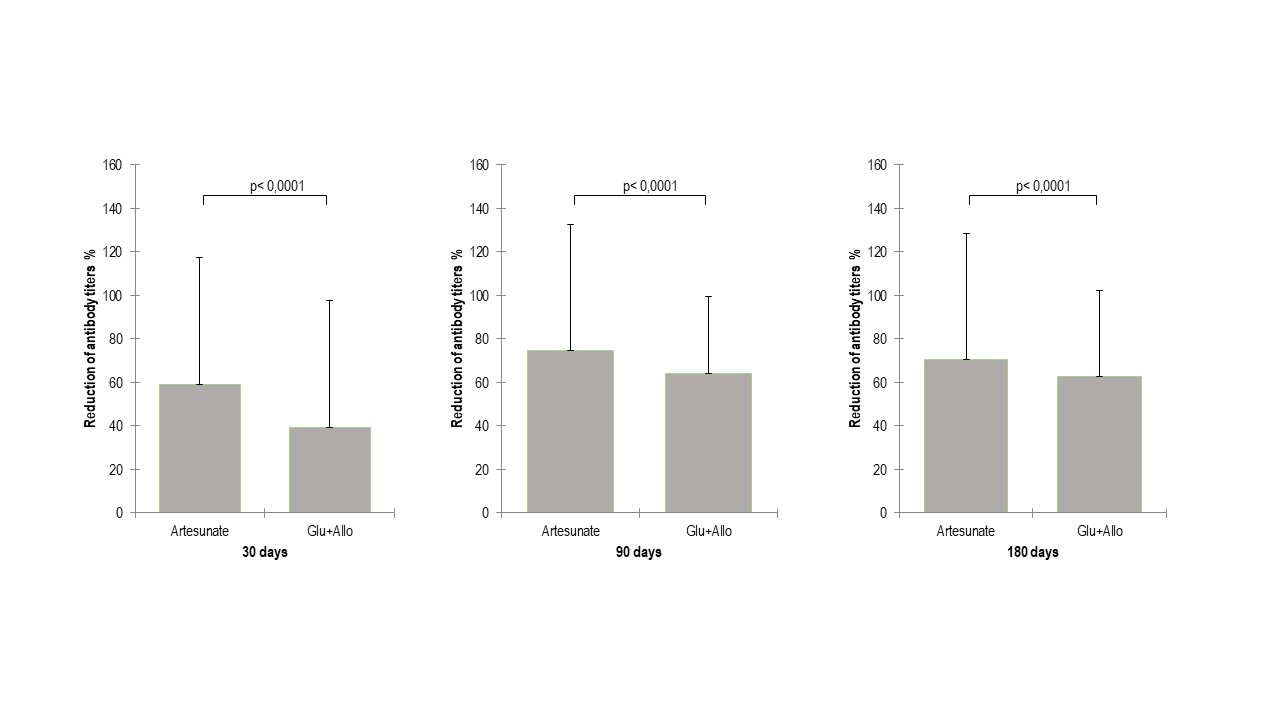

Supplement: S1 Fig — Data are reported as the mean ± SD, artesunate versus Glucantime/allopurinol (Mann-Whitney test) 30-, 90- and 180-days posttreatment. (TIF) [file pntd.0008947.s001.tif]
