# Supplementary material for: Potential of Artesunate in the treatment of visceral leishmaniasis in dogs naturally infected by Leishmania infantum: Efficacy evidence from a randomized field trial
Source: PLoS Negl Trop Dis. 2020 Dec 18;14(12):e0008947. doi: 10.1371/journal.pntd.0008947 (PMC7781483; doi:10.1371/journal.pntd.0008947)
Supplement: S1 Table — (DOCX) [file pntd.0008947.s002.docx]

**Fiche of follow-up Day- 0, 30, 90 or 180**

- **Date: Dog ID :**
- **Witness Leishmania Test: + -**
- Commune: Village: Wilaya:
- Name of the owner:
- Mobile:
- Nam of the veterinarian:
- Dog identification:

| **Breed** | **Sex** | **Age** | **Origin** | **Activity** |
| --- | --- | --- | --- | --- |
|  |  |  |  |  |

- Observed signs (complete the table below) :

| **Signs** | **frequency** | | | |
| --- | --- | --- | --- | --- |
|  | **Absent**  **0** | **Mild**  **+** | **moderate**  **++** | **Severe**  **+++** |
| **At the general condition level** |  |  |  |  |
| Amaigrissement |  |  |  |  |
| Anorexia |  |  |  |  |
| Abatement |  |  |  |  |
| Anemia |  |  |  |  |
| Hyperthermia |  |  |  |  |
| **At the cutaneous level** |  |  |  |  |
| Localized allopecia |  |  |  |  |
| Squamosis |  |  |  |  |
| Hyperkeratosis |  |  |  |  |
| Onychogriffosis |  |  |  |  |
| Ulcerations |  |  |  |  |
| **At the ophthalmic level** |  |  |  |  |
| Depilation in glasses |  |  |  |  |
| Conjunctivitis |  |  |  |  |
| **At the oral level** |  |  |  |  |
| Erosions |  |  |  |  |
| **At the skeletal level** |  |  |  |  |
| Arthritis, synovitis |  |  |  |  |
| Diffuse posterior train pain |  |  |  |  |
| **Divers** |  |  |  |  |
| Adenopathy |  |  |  |  |
| Splenomegaly |  |  |  |  |
| Epistaxis |  |  |  |  |
| Renal disorders |  |  |  |  |
| Chronical diarrhea |  |  |  |  |
| Others (define) |  |  |  |  |

**Treatment:**

**-Remarks**:

**-Comments** (in particular on the location of lesions and reactions to treatments):
